# Delayed mechanical ventilation with prolonged high-flow nasal cannula exposure time as a risk factor for mortality in acute respiratory distress syndrome due to SARS-CoV-2

**DOI:** 10.1007/s11739-022-03186-4

**Published:** 2023-02-15

**Authors:** Viviana Yiset López-Ramírez, Oscar Orlando Sanabria-Rodríguez, Santiago Bottia-Córdoba, Oscar Mauricio Muñoz-Velandia

**Affiliations:** 1 Pontificia Universidad Javeriana, Cra. 7 # 40-62, Piso 5, Bogotá, Colombia; 2 Pontificia Universidad Javeriana—Hospital Universitario San Ignacio, Bogotá, Colombia

**Keywords:** Coronavirus infections, Acute respiratory distress syndrome, Noninvasive ventilation, Pandemics, Mortality

## Abstract

In a high proportion of patients, infection by COVID-19 progresses to acute respiratory distress syndrome (ARDS), requiring invasive mechanical ventilation (IMV) and admission to an intensive care unit (ICU). Other devices, such as a high-flow nasal cannula (HFNC), have been alternatives to IMV in settings with limited resources. This study evaluates whether HFNC exposure time prior to IMV is associated with mortality. This observational, analytical study was conducted on a historical cohort of adults with ARDS due to SARS-CoV-2 who were exposed to HFNC and subsequently underwent IMV. Univariate and multivariate logistic regression was used to analyze the impact of HFNC exposure time on mortality, controlling for multiple potential confounders. Of 325 patients with ARDS, 41 received treatment with HFNC for more than 48 h before IMV initiation. These patients had a higher mortality rate (43.9% vs. 27.1%, *p*: 0.027) than those using HFNC < 48 h. Univariate analysis evidenced an association between mortality and HFNC ≥ 48 h (OR 2.16. 95% CI 1.087–4.287. *p*: 0.028). Such an association persisted in the multivariable analysis (OR 2.21. 95% CI 1.013–4.808. *p*: 0.046) after controlling for age, sex, comorbidities, basal severity of infection, and complications. This study also identified a significant increase in mortality after 36 h in HFNC (46.3%, *p*: 0.003). In patients with ARDS due to COVID-19, HFNC exposure ≥ 48 h prior to IMV is a factor associated with mortality after controlling multiple confounders. Physiological mechanisms for such an association are need to be defined.

## Introduction

The COVID-19 pandemic has challenged healthcare systems around the world to provide optimal medical care for all patients infected by SARS-CoV-2, including those who developed acute respiratory distress syndrome (ARDS) and required invasive mechanical ventilation (IMV) and admission to intensive care unit (ICU) [1–4]. In the most critical moments of the pandemic, availability of resources has been limited, which has led to use of oxygenation strategies other than IMV [5–9].

Recent guidelines of the European respiratory society suggest high-flow nasal cannula (HFNC) over conventional oxygen therapy (COT) and noninvasive ventilation (NIV) in hypoxaemic acute respiratory failure [10]. However, systematic reviews on the management of patients with HFNC have limitations related to the high heterogeneity and the small numbers of patients included. A review concludes that HFNC reduced the need of orotracheal intubation (OTI) (OR 0.62. 95% CI 0.38–0.99) and ICU mortality (OR 0.47. 95%CI 0.24–0.93) [11]. Other reviews indicated that HFNC may reduce OTI need and IMV initiation (Relative risk 0.85. 95% CI 0.74–0.99; Number needed to treat: 23. 95% CI 13–333) with no changes in mortality (RR 0.94 95%CI 0.67–1.31) [12, 13]. Also, by improving oxygenation, use of HFNC may create a sensation of security that may delay endotracheal intubation. Delay to initiate OTI would expose the patient to a respiratory effort leading to patient self-inflicted lung injury (P-SILI) through a mechanism similar to ventilation-induced lung injury (VILI). The P-SILI may increase mortality in ARDS [14–19].

It is not clear whether a longer HFNC exposure and consequent delay to endotracheal intubation worsens prognosis in patients with ARDS. Two previous studies by Kang [20] and Miller [21] suggested that mortality is higher in patients receiving delayed OTI (≥ 48 h) after HFNC failure. Conversely, a study in Atlanta, Georgia (USA.) conducted on a retrospective cohort of patients receiving delayed switch to IMV after HFNC use found no difference in mortality, ICU length of stay, or IMV duration [22]. The objective of this study is to determine whether a delayed endotracheal intubation after a prolonged time in HFNC is a risk factor for mortality in adults with severe pneumonia and ARDS due to SARS-CoV-2. The study was conducted in a cohort of patients admitted to the ICU of a reference hospital in Colombia.

## Methods

This observational, analytical study was conducted from June 2020 to February 2022 on a historical cohort of patients with severe pneumonia and ARDS due to SARS-CoV-2. Patients in the cohort received initial treatment with HFNC and subsequently underwent IMV in the ICU of Hospital Universitario San Ignacio in Bogotá D.C., Colombia. The HFNC was started because the patient’s clinical condition predicted a good response to this therapy or because mechanical ventilators were not available. Inclusion criteria were:

- Age above 18 years
- SARS-CoV-2 infection confirmed by RT-PCR, antigen, or FilmArray
- Severe pneumonia defined by the Colombian consensus for management of SARS-CoV-2 infection criteria: respiratory rate > 30 rpm, respiratory distress, or SaO2 < 90% at ambient air [2]
- Mild, moderate, or severe ARDS by Berlin criterion. The study did not consider the PEEP criterion, since clinicians considered ARDS diagnosis prior to IMV use [23].
- Ventilatory support, initially with HFNC and subsequently with IMV. The switch to IMV could be due to a failure of the HFNC or due to the availability of IMV after it was not initially available.

Exclusion criteria were referral from or to another institution, coinfection by other viruses, such as influenza A and/or B, syncytial respiratory virus, or adenovirus, and prior SARS-CoV-2 infection requiring admission to ICU. The research project was approved by the institutional ethics committee (Act No. 16/2021) and was performed in accordance with the ethical standards laid down in the 1964 Declaration of Helsinki and its later amendments.

The authors screened the ICU database for patients who were admitted with diagnosis of SARS-CoV-2 infection. Then, they reviewed the electronic clinical records in the institutional platform SAHI^®^ to verify inclusion and exclusion criteria. A standard form was used to collect data that included age, sex, weight, height, comorbidities, vaccination state, Charlson comorbidity index [24], and Pittsburgh priority score in COVID-19 [25]. The study also collected information on the disease’s clinical presentation: time with symptoms at initial consultation, laboratory results at hospital admission, and the Sequential Organ Failure Assessment (SOFA) index at ICU admission [26]. Records also included respiratory rate, ROX index [27], arterial blood gas measurement, and characteristics of pulmonary mechanics (compliance, compliance pressure, plateau pressure, PEEP, and tidal volume) at HFNC initiation and IMV initiation. The study also included therapeutical aspects, such as use of steroids, neuromuscular relaxants, and pronation. We planned to record standard oxygen therapy (SOT) time prior to arrival or in the emergency department prior to HFNC or MVI, but the information was not recorded consistently. Assessed outcomes were length of IMV, length of ICU and hospital stay, bacterial pneumonia, bacteremia, and death.

The HFNC failure was defined according with international guidelines [10], by a team that included the attending physician and two or more additional physicians who considered the clinical status of the patient and resources availability. A cut-off threshold of 5.9 was used for ROX index, as suggested for COVID-19 patients [27]. In Colombia, the COVID-19 historic case report defined four epidemiological peaks: (1) July to October 2020, (2) November 2020 to March 2021, (3) April to September 2021 and, (4) October 2021 to February 2022. Such a distinction allowed a sensitivity analysis based on the period when the patient received healthcare.

Calculation of sample size used the concept of “event by variable of interest” suggested by Freeman [28]. In addition to the studied variable (time between HFNC initiation and IMV initiation), the study included ten events of mortality for each confounding variable assessed, for a total of 12 confounders. Assuming an expected mortality of 40% [29], the required sample size was 325 patients.

The study presents continuous variables with central tendency and dispersion measures of: (i) average and standard deviation for normally distributed variables (ii) median and interquartile range for variables not fulfilling that assumption. Report of categorical variables used absolute numbers and percentages. Comparability of delayed endotracheal intubation by groups (HFNC use more or less than 48 h) used *t* test, Mann–Whitney *U* test, or chi-square test, by type of variable. Selected cut-off point for HFNC exposure time was 48 h, as it has been reported in the literature as a prognosis factor [19, 20]. Further, univariable and multivariable logistic regression analyses for mortality, included delayed IOT and previously defined confounders, including previously diagnosed respiratory diseases. Selection of variables for the final model used stepwise backward elimination. A *p* value < 0.05 was considered statistically significant. Statistical analysis used the IBM SPSS Statistics 25 software.

## Results

From June 2020 to February 2022, the ICU of Hospital Universitario San Ignacio admitted 1,075 patients and 325 fulfilled inclusion criteria for this study. Table 1 presents basal characteristics of included patients. The average patient age was 59 ± 13.04, and 74% were males. The principal comorbidities were obesity or overweight, high blood pressure, tobacco use, type 2 diabetes mellitus, and hypothyroidism. The main previously diagnosed respiratory disease was COPD (9%). Charlson comorbidity and SOFA indexes at ICU admission were high. By the Pittsburgh priority score, 55% of patients had a high priority for critical-care resource allocation.

**Table 1:** Patients basal characteristics and outcomes

Characteristic	All (*n* = 325)	HFNC < 48 h (*n* = 284)	HFNC ≥ 48 h (*n* = 41)	*p* value
Age, years, average (SD)	59 (± 13.04)	59 (± 13.44)	64 (± 8.73)	0.018
Sex, males *n* (%)	240 (74)	203 (72)	37 (90)	0.011
Comorbidities *n* (%)				
Overweight or obesity (*n* = 318)	234 (72)	210 (74)	24 (59)	0.068
High blood pressure	117 (36)	105 (37)	12 (29)	0.337
Tobacco use	65 (20)	59 (21)	6 (15)	0.358
Type-2 diabetes mellitus	60 (18)	54 (19)	6 (15)	0.499
Hypothyroidism	48 (14)	39 (14)	9 (22)	0.166
Chronic obstructive pulmonary disease	29 (9)	26 (9)	3 (7)	0.700
Coronary disease	20 (6)	16 (6)	4 (10)	0.305
Biomass exposure	17 (5)	13 (5)	4 (10)	0.164
Active cancer	16 (5)	13 (5)	3 (7)	0.448
Heart failure	14 (4)	9 (3)	5 (12)	0.008
Charlson comorbidity index ≥ 3 *n* (%)	124 (38)	108 (38)	16 (39)	0.902
SOFA at ICU admission ≥ 2 *n* (%)	319 (98)	279 (98)	40 (98)	0.763
Vaccinated, *n* (%)	26 (8)	22 ( 7.7)	4 ( 9.7)	0.657
Laboratory parameters—median (IQR)				
Hemoglobin—g/dL	15.5 (14.3–16.4)	15.5 (14.3–16.4)	15.2 (14.2–16.2)	0.542
Platelets—/µL	209,700 (158,950–270,850)	210,800 (160,300–267,250)	208,800 (151,800–285,800)	0.960
Lymphocytes—/µL	800 (600–1100)	800 (600–1100)	800 (500–1100)	0.515
D-Dymer—ng/mL (*n* = 320)	768 (506–1201)	766 (506–1178)	773 (484–1514)	0.845
Creatinine—mg/dL	0.94 (0.77–1.10)	0.93 (0.76–1.09)	1.01 (0.79–1.17)	0.182
LDH—U/L (*n* = 322)	414 (334–529)	410 (334–525)	434 (339–571)	0.709
PCR—mg/dL (*n* = 319)	14.28 (9.7–21.21)	14.3 (9.7–21.5)	14.8 (10.0–21.0)	0.928
Other treatments—*n* (%)				
Steroid*	321 (99)	280 (99)	41 (100)	0.444
Neuromuscular relaxant†	274 (84)	238 (84)	36 (88)	0.510
Pronation	245 (75)	217 (76)	28 (68)	0.259
Complications and outcomes—*n* (%)				
Bacteremia^‡^	125 (39)	113 (40)	12 (29)	0.196
IMV-associated bacterial pneumonia^‡^	98 (30)	85 (30)	13 (32)	0.817
Acute kidney injury^∫^	97 (30)	86 (30)	11 (27)	0.652
Pulmonary embolism	36 (11.1)	29 (10.2)	7 (17.1)	0.191
Time with symptoms at consultation- days, median (IQR)	7 (5–10)	7 (5–10)	7 (4–10)	0.405
Hospital stay—days, median (IQR)	20 (14–27)	20 (14–27)	23 (14–26)	0.880
ICU stay—days, median (IQR)	12 (7–18)	12 (8–19)	10 (6–15)	0.015
Time on IMV—days, median (IQR)	10 (6–15)	11 (7–16)	8 (5–13)	0.025
Death	95 (29)	77 (27)	18 (44)	0.027

*HFNC* high-flow nasal cannula, *SD* standard deviation, *ICU* intensive care unit, *LDH* lactate dehydrogenase, *CRP* C-reactive protein, *IMV* invasive mechanical ventilation, *IQR* interquartile range, *SOFA* sequential organ failure assessment

*RECOVERY study [49] recommends 6 mg of intravenous dexamethasone once a day for 10 days

^†^ACURASYS study [50] recommends cisatracurium for 48 h. Rocuronium was occasionally used, based on availability

^‡^The study assumed IMV-associated bacteremia or pneumonia when conditions were recorded in a clinical history and supported by clinically significant microbiological isolation in blood that required antibiotic coverage

^∫^The study assumed acute kidney injury when the diagnosis was recorded in a clinical history and supported by KIDGO criteria on nitrogenous compound elevation and urine output abnormality [51]

Outstanding laboratory parameters include low lymphocyte count (median, 800/µL), as well as elevated D-dimer, LDH, and CRP. Almost all patients received steroids, and a high percentage underwent neuromuscular relaxation (84%) and pronation (75.4%). Complications, such as bacteremia, ventilator-associated bacterial pneumonia, and kidney injury, are presented in almost one-third of patients. Median ICU stay was 12 days, hospital stay 20 days, IMV time 10 days, and mortality 29.2%. Comparison between patients with early or delayed IMV (HFNC for more or less than 48 h) showed that patients in the first group were older (64 vs. 59 years, *p*: 0.018), mostly males (90.2 vs. 71.5%, *p*: 0.011), and had a higher mortality rate (43.9 vs. 27.1%, *p*: 0.027) (Table 1).

Ventilatory parameters at HFNC initiation showed a median respiratory rate of 24 rpm, a high percentage of patients (46.9%) with a ROX index > 5.9 predicting “low risk of intubation,” and a moderate oxygenation disorder in all patients. Initial HFNC setting used high parameters, considering that the maximum provided by this system is an oxygen flow of 60 L/min and a FiO_2_ of 100%. At IMV initiation, there was no significant change in the respiratory rate, but the percentage of patients with ROX score > 5.9 was lower. Also, oxygenation disorder was severe, and the parameters were established for protective ventilation. Twenty-four hours after the start of IMV, blood gas parameters showed a drop in pH at the expense of an increase in PaCO_2_ and a slight increase in PaO_2_ and PaO_2_:FiO_2_. Those parameters, however, remained within the range defined for a moderate oxygenation disorder (Table 2). There were no significant differences in arterial blood gas parameters between patients in HFNC for more or less than 48 h.

**Table 2:** Patients ventilatory mechanics

Characteristic—median (IQR)	All (*n* = 325)	HFNC < 48 h (*n* = 284)	HFNC ≥ 48 h (*n* = 41)	*p* value
Clinical condition at HFNC initiation				
Respiratory rate—rpm	24 (21–28)	24 (21–28)	24 (22–28)	0.696
ROX index > 5.9—*n* (%)	152 (46.9)	130 (45.9)	22 (53.6)	0.356
pH	7.46 (7.43–7.48)	7.46 (7.43–7.48)	7.46 (7.43–7.48)	0.884
PaO_2_—mmHg	67 (61–78)	69 (60–78)	67 (62–80)	0.615
PaCO_2_—mmHg	30 (27–33)	30 (27–33)	30 (27–32)	0.519
PaO_2_:FiO_2_ ratio—mmHg, average (SD)	126 (± 44)	126 (± 44)	114 (± 31)	0.089
HFNC settings				
FiO_2_—%, average (SD)	77 (± 13.97)	77 (± 13.86)	74 (± 14.58)	0.200
Flow—L/min, average (SD)	55 (± 4.72)	56 (± 4.17)	55 (± 7.49)	0.178
Clinical condition at IMV initiation				
Respiratory rate-rpm	25 (22–30)	26 (22–30)	24 (20–28)	0.207
ROX index > 5.9—no. (%)	57 (17.6)	48 (17)	9 (21.9)	0.442
PaO2:FiO_2_—mmHg	90 (74–110)	90 (74–110)	87 (72–116)	0.315
Time in HFNC—hours	16 (8–29)	14 (7–23)	81 (63–119)	< 0.001
Static compliance- ml/cmH_2_O	40 (32–46)	40 (32–46)	40 (35–47)	0.454
Plateau pressure—cmH_2_O	22 (20–24)	22 (20–24)	22 (20–24)	0.502
Compliance pressure—cmH_2_O	10 (9–12)	10 (9–12)	11 (9–12)	0.237
Optimal PEEP—cmH_2_O	12 (10–12)	12 (10–12)	10 (10–12)	0.415
Tidal volume—mL/kg	7.0 (6.5–7.0)	7.0 (6.5–7.0)	7.0 (6.5–7.0)	0.556
Clinical condition 24 h post IMV				
pH	7.36 (7.31–7.41)	7.36 (7.31–7.41)	7.34 (7.27–7.41)	0.300
PaO_2_—mmHg	79 (73–87)	79 (73–87)	78 (71–87)	0.668
PaCO_2_—mmHg	46 (40–53)	45 (40–53)	49 (43–59)	0.023
PaO_2_:FiO_2_ ratio—mmHg	168 (128–203)	168 (134–203)	168 (111–204)	0.315

*HFNC* high-flow nasal cannula, *IQR* interquartile range, *SD* standard deviation, *IMV* invasive mechanical ventilation

The univariate analysis showed an association between mortality and delayed IMV (exposure to HFNC ≥ 48 h) (OR 2.16. 95% CI 1.087–4.287. *p*: 0.028), age, history of high blood pressure, type 2 diabetes, chronic obstructive pulmonary disease (COPD), heart failure, acute kidney injury during ICU stay, and elevated Charlson score and SOFA indexes. In the multivariable analysis, the association between mortality and delayed IMV (OR 2.21. 95% CI 1.013–4.808. *p*: 0.046), age, COPD, and acute kidney injury was also significant (Table 3).

**Table 3:** Mortality-predicting factors in COVID-19 patients according with early or delayed endotracheal intubation after HFNC use

Variable	Univariable analysis			Multivariable analysis		
	Raw OR	95% CI	*p* value	Adjusted OR	95% CI	*p* value
HFNC exposure time ≥ 48 h	2.16	1.087–4.287	0.028	2.21	1.013–4.808	0.046
Age	1.07	1.043–1.091	< 0.001	1.05	1.021–1.073	< 0.001
Sex, male	1.75	0.970–3.160	0.059	–	–	–
High blood pressure	1.97	1.210–3.223	0.006	–	–	–
Type-2 diabetes mellitus	1.82	1.016–3.265	0.044	–	–	–
Chronic obstructive pulmonary disease	3.38	1.556–7.344	0.002	2.15	0.868–5.345	0.098
Chronic kidney disease	2.48	0.608–10.143	0.205	–	–	–
Heart failure	4.71	1.535–14.449	0.007	–	–	–
Charlson comorbidity index ≥ 2	4.67	2.601–8.379	< 0.001	–	–	–
SOFA ≥ 4	2.13	1.128–4.040	0.020	–	–	–
Acute kidney injury	6.15	3.633–10.426	< 0.001	5.19	2.925–9.226	< 0.001
Pulmonary embolism	1.24	0.593–2.597	0.567	–	–	–
Vaccination	2.23	0.991–5.022	0.053	–	–	–

*HFNC* high-flow nasal cannula, *IMV* invasive mechanical ventilation, *OR* odds ratio, *CI* confidence interval, *SOFA* sequential organ failure assessment

*R*^2^ = 0.1938

Analysis of HFNC exposure time showed that mortality was significantly higher after 36 h (46.3%, *p*: 0.003).

**Fig. 1:**
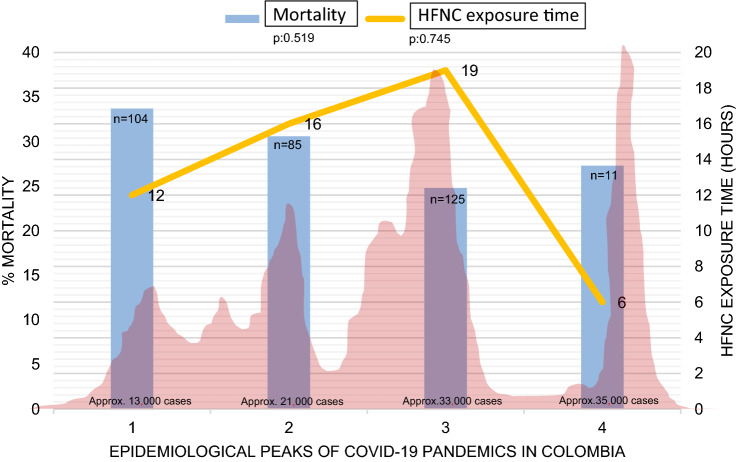
Mortality and HFNC exposure time by epidemiological peaks of COVID-19 pandemic in Colombia. The *pink graphic* represents the historical report of COVID-19 cases by the National Institute of Health of Colombia, comprising four epidemiological peaks. First, July–October 2020. Second, November 2020–March 2021. Third, April–September 2021. Fourth, October 2021–February 2022. The number of cases reached 35,000 per day in the most critical moments. The *blue bars* show mortality in each peak, 33.7%, 30.6%, 24.8%, and 27.3%, respectively, with no statistically significant difference among peaks. The *yellow line* represents the median HFNC exposure time prior to IMV. The line shows prolongation in the third peak, since it was higher and lengthier. The HFNC exposure time prolongation was not statistically significant (Color figure online)

Figure 1 presents mortality of patients on HFNC > 36 h in each epidemiological peak (blue bars). There was no statistically significant difference among the four peaks. Median HFNC exposure time prior to IMV was lower in the fourth peak (yellow line). That variable did not reach the 36-h threshold in any peak.

## Discussion

The present study suggests that delayed endotracheal intubation after a prolonged HFNC exposure time is a risk factor for mortality in patients with ARDS due to COVID-19. The study controlled multiple confounders, such as age, sex, basal comorbidities, clinical severity at admission, and the appearance of complications such as kidney failure. We also found that mortality rates are significantly higher after HFNC ≥ 36 h.

Other mortality studies were conducted during the COVID-19 pandemic on patients undergoing HFNC and requiring further IMV. A study at Temple University Hospital in Philadelphia (Pennsylvania, USA) [30] reported a mortality of 35.1%. Data from Delbove [31] were comparable. Panadero [32] reported a mortality of 42.8%, and Alshahrani [33] as high as 52%. These rates are discretely higher than the mortality in the present study (29%), probably because our population had a lower frequency of comorbidities and a shorter time between symptoms onset and consultation. Chandel [34] found an association between HFNC failure and mortality (adjusted OR 2.13. 95%CI 0.80–5.62. *p*: 0.13), regardless of the exposure time. Baek [35] reported similar findings (adjusted OR 4.75. 95% CI 1.118–20.236. *p*: 0.035).

In a study conducted prior to the COVID-19 pandemic in South Korea, Kang [20] classified HFNC failure according to IMV requirement in early (within 48 h of HFNC) and late (after 48 h) groups. The author found higher mortality in patients with late HFNC failure (39.2 vs. 66.7%, *p*: 0.001). This group also had disadvantages in terms of extubation timing and ventilator-free days [20]. Baek reported similar findings in COVID-19 patients [35], with mortality at 38% for early HFNC failure and 65% for late HFNC failure (*p*: 0.041).

Data in the present study suggest that mortality increases with a delayed switch to IMV after a prolonged time in HFNC. This may be attributed to mechanisms of P-SILI [36–39]. Noticeably, HFNC exposure time did not influence pulmonary mechanics assessed at IMV initiation by static compliance, plateau pressure, and compliance pressure. It is possible that other mechanisms, including biotrauma in the non-ventilated patient, lead to death. Unfortunately, due to its retrospective nature, this study did not include cytokine measurement. Assessment of such a hypothesis requires prospective studies.

We did not measure physiological variables to assess respiratory effort and its impact in transpulmonary pressure. However, other studies have shown that compared with the non-COVID-19 group, patients with COVID-19 before NIV showed lower values of inspiratory effort assessed by esophageal pressure [40]. This relatively low inspiratory effort did not increase dynamic transpulmonary conduction pressure, a behavior different from that of patients without COVID-19 [41]. Additionally, unphysiological values of esophageal pressure swings in COVID-19 patients have been reported [42]. Another study used a computational cardiopulmonary physiology simulator to measure the presentation of P-SILI according to the patient’s respiratory effort. Conversely, it suggested changes in pleural pressure, transpulmonary pressure, mechanical pressure, and compliance pressure as the tidal volume and respiratory rate increased due to respiratory effort [43].

Early intubation is not the rule for all patients with ARDS, and studies support the use of HFNC as a strategy to reduce the IOT rate [44–46]. This study suggests that close clinical monitoring of patients is required to find the right moment to switch to IMV, proposing not to exceed the relative safety time of 36 h in HFNC.

We found that HFNC exposure time varied discretely among peaks of the COVID-19 pandemic. This was associated with varying availability of healthcare resources. Mortality, however, did not vary among peaks, probably because the 36-h HFNC threshold was not surpassed. In fact, HFNC was a relatively safe strategy for patients’ support, while IMV was accessible. The HFNC also assured an optimal use of resources, as demonstrated by Gershengorn [47].

All patients in this study ended up requiring IMV. At HFNC initiation, however, a large percentage of them had a high enough ROX index to predict HFNC “success” [48]. Hu [49] reported similar findings, with a ROX index of 6.4 at 2 h of HFNC [49]. Differently, Panadero [32] and Alshahrani [33] reported that HFNC failure had a ROX index < 3.7 at HFNC initiation. Chandel [34] also found lower ROX indexes than in this study at 2, 6, and 12 h of HFNC initiation and when IMV support was decided. These data question the use of the ROX index as a tool to predict success with HFNC in patients with COVID 19 and suggest that if it is used, a higher cut-off value should be considered [27, 50].

Some recent studies have found that vaccinated patients have better clinical course and outcomes as compared to the unvaccinated population [51]; in our study, a small proportion of patients (8%) had been vaccinated before requiring IMV. Our data suggest that delayed endotracheal intubation has a similar impact in the vaccinated and unvaccinated groups.

Among strengths of this study, standardization of parameters for HFNC and IMV initiation is conspicuous. Such standardization aims for protective conditions in mechanical ventilation, even though a 6 ml/kg tidal volume that the ARDS guidelines recommend was not used with the majority of patients [52]. Availability of all data required for the study is also remarkable. This availability results from systematic recording of the respiratory mechanics in the clinical history by trained staff. The authors, however, acknowledge that the retrospective nature is a limitation of the study; this prevented the evaluation of the time in SOT prior to IMV or HFNC. A second limitation is the lack of evaluation of the physiological mechanisms in the association between HFNC and possible P-SILI. Future studies are needed to better understand the pathophysiology of this association. Finally, the external validity of the present findings needs assessment. Data in this study, however, may be useful for patients with ARDS with causes other than COVID-19, especially considering that its unlikely clinics will see a number of patients with ARDS as high as seen in COVID pandemic available for research.

## Conclusions

Results in this study suggest that delayed endotracheal intubation after a HFNC exposure ≥ 48 h increases patients mortality risk independently of comorbidities and disease severity at admission. The results also suggest that the mortality risk increase may be significant after 36 h of HFNC. Physiological mechanisms for this association need further prospective studies.

## Data Availability

Data is available from the authors upon request.
